# Is the walk and trot efficiency a hereditary trait in primitive Konik Polski horses?

**DOI:** 10.7717/peerj.21369

**Published:** 2026-05-27

**Authors:** Marta Siemieniuch-Tartanus, Iwona Tomczyk-Wrona, Dawid Tobolski

**Affiliations:** 1 Department of Large Animal Diseases and Clinic, Warsaw University of the Life Science, Warsaw, Poland; 2 Department of Farm Animal Biodiversity Conservation and Horse Breeding, National Research Institute of Animal Production, Cracov-Balice, Poland

**Keywords:** Gait analysis, Genetic trend, Hereditary traits, Primitive breed, Longitudinal study, Horse

## Abstract

**Background:**

Performance testing is a critical tool for achieving genetic progress, yet its long-term effectiveness in primitive breeds under conservation programs is poorly documented. The Konik Polski horse breeding program aims to improve utility while preserving primitive characteristics.

**Methods:**

This retrospective study analyzed a 25-year longitudinal dataset (*n* = 1,608) from the official Field Performance Test to quantify genetic trends in gait efficiency, using nonparametric methods to assess the effects of test type, year, and age on stride length and speed at the walk and trot. To estimate heritability, a single-trait sire model and a linear mixed model were fitted.

**Results:**

A significant positive trend was observed for walk stride length, which increased by approximately 10 cm under saddle and 19 cm in harness over the study period (*p* < 0.001). In contrast, trot stride length showed high variability but no consistent trend, whereas trot speed declined. A strong negative correlation between trot stride length and trot time (*ρ* =  − 0.51) confirmed that longer strides were associated with faster movement, which is a desirable trait. The analysis revealed moderate heritability for walk stride length (*h*^2^ = 0.277). Genealogical line accounted for a negligible proportion of the total phenotypic variance for most traits, and age at testing did not significantly influence performance. Walk- and trot-related performance traits were not highly heritable in Konik Polski horses. Overall, the results indicate that the level of preparation is the most critical factor.

## Introduction

Effective breeding programs designed for rapid and continuous genetic progress rely on a clear definition of breeding goals and rigorous selection for traits that align with these objectives. Young-horse performance tests are central to this process, providing crucial data for the genetic evaluation of young horses and their parents, often using the Best Linear Unbiased Prediction (BLUP) animal model or other statistical models (Torzynski & Szwaczkowski, 1999; Ricard & Touvais, 2007; Rustin et al., 2009; Stewart et al., 2012; Sun et al., 2009; Viklund et al., 2011; Novotna et al., 2022; Vosgerau et al., 2022; Ziadi et al., 2025). These tests also serve as a vital tool for identifying talented individuals for sport. The rate of genetic progress depends on reasonably high heritability for the recorded traits and adequate selection intensity (Thorén Hellsten et al., 2006; Lewczuk et al., 2026). For many sport horse populations, particularly Warmbloods, a studbook’s breeding success is measured by the competitive performance of its horses at the highest level. Because elite competition results are a primary objective for most sport horse breeds, tests conducted at a young age must exhibit a strong positive genetic correlation with later career performance. Warmblood performance tests, for example, evaluate a comprehensive set of traits, including character, temperament, trainability, free jumping, jumping under rider, gait quality (walk, trot, and canter), rideability, and overall potential for dressage and jumping (Koenen, Aldridge & Philipsson, 2004; Viklund et al., 2011; Lewczuk et al., 2026). In contrast, other breeds are selected for different specializations. Purebred Arabians and Thoroughbreds undergo performance trials on racetracks to assess speed (Klecel, Drobik-Czwarno & Martyniuk, 2021). Ultimately, performance tests are specifically developed for each breed to ensure that individuals meet established standards, with evaluations tailored to the horse’s distinct anatomy, physiology, and intended use (Lewczuk, 2015).

The Konik Polski horse (KPH) is a primitive breed whose phenotype has been shaped more by natural pressures than by intensive artificial selection. Although the breed’s recorded history is relatively short, KPHs have been used for decades in landscape management and the maintenance of grassland ecosystems. Initial conservation breeding efforts aimed at restoring the Tarpan were established in a reserve in the Białowie.za Forest in 1936 on the initiative of Professor Tadeusz Vetulani. Although the theory that the KPH is a direct descendant of the Tarpan has been widely accepted for decades (Pasicka, 2013), recent years have cast doubt on this hypothesis (Lovasz, Fages & Amrhein, 2021). Classification of mtDNA haplotypes across individual maternal lineages revealed that they belong to six haplogroups (A, B, E, J, G, R), with a clear predominance of haplotypes most commonly found in Asian horse populations. Analysis of the Y chromosome showed that most paternal lineages of the KPH shared haplotypes with cold-blooded horse breeds and also indicated possible interbreeding among breeds (Musiał et al., 2024). After the war, the surviving horses formed the basis for reestablishing the conservation program at a new reserve in Popielno, which began in 1955. Alongside this reserve-based conservation, which maintains horses in semi-feral conditions, formal stud breeding of KPHs also developed (Górecka-Bruzda et al., 2020). Currently, approximately 4% of the KPH population is managed in semi-free conditions within reserves in Poland (Niedbalska, 2012).

The use of KPHs in conservation aligns with a recent increase in interest across Europe in using large herbivores to revitalize and manage green spaces (Korzekwa et al., 2023). Nature conservation initiatives, such as Rewilding Europe (https://rewildingeurope.com), actively promote grazing by robust local horse breeds as a key management tool (Linnartz & Meissner, 2014). Within rewilding projects that aim to rebuild self-sustaining, complex ecosystems, horses contribute to the restoration of diversified herbivory, which benefits overall ecosystem functioning and biodiversity (Svenning, Buitenwerf & Le Roux, 2024). KPHs have proven remarkably efficient at shaping floristic diversity (Chodkiewicz, 2020) and enhancing faunal richness and abundance, particularly in bird communities (Köhler, Hiller & Tischew, 2016; Lovasz, Korner-Nievergelt & Amrhein, 2024).

While the reserve population fulfills a crucial ecological function, horses managed under semi-feral conditions are not subjected to performance evaluation. Consequently, the establishment of formal stud breeding for KPHs prompted the need to define a utilitarian purpose for the breed beyond its role in landscape maintenance. The anatomical features of the KPH limit its suitability for elite competitive sports. Nevertheless, horses of this breed are increasingly used for recreational riding, educational or therapeutic purposes, equestrian tourism, and low-level sports because of their relatively good health, low maintenance costs, and gentle, balanced temperament compared with Warmblood horses (Hendricks, 2007). The performance tests for this breed were therefore designed to align with the dual objectives of the official breeding program: maintaining the primitive phenotype while simultaneously improving performance traits. This approach, however, contrasts with an alternative viewpoint in conservation breeding, which holds that no selection should be applied to primitive breeds in order to preserve their genetic makeup unchanged for future generations (Jezierski, 1990; Meuwissen, 1997; Wellmann, Hartwig & Bennewitz, 2012).

Genetic improvement within the KPH population is achieved through both environmental management and structured breeding programs. In contrast to Warmblood breeds with longer histories of performance-based selection, breeding efforts in KPHs have for many years focused primarily on preserving specific phenotypic traits, such as the characteristic mouse-grey coat with a dorsal stripe and the absence of white markings. A systematic focus on improving performance traits began around the year 2000 with the introduction of annual field performance tests (FPTs), initially mandatory only for stallions. These tests provide objective data to assess an individual’s performance potential based on strength, speed, and endurance.

Currently, these FPTs are mandatory for both sexes; stallions must complete the test between three and five years of age, whereas mares are required to do so within four years of registration in the studbook. Furthermore, to be included in the optional genetic resources conservation program (GRCP), which provides subsidies to breeders, mares must successfully complete an FPT for initial entry. Despite substantial population growth, the KPH is still classified by the food and agriculture organization (FAO) as a breed under a conservation program requiring population monitoring (Chodkiewicz, 2020). In such small populations, breeding strategies must be carefully designed to mitigate the risks of increased inbreeding and the loss of valuable genetic diversity. This is particularly important in this breed because the initial population consisted of 35 mares and 6 stallions, which established the breed’s genealogical lines. Currently, only 16 mare lines remain in breeding, whereas the other 19 lines have become extinct. These 16 female lines are represented very unevenly (Jaworski, 1997). The situation is further complicated by the fact that, according to recent studies, female lineages do not fully correspond to genetic lineages as determined by mitochondrial DNA testing (Cieslak et al., 2017; Musiał et al., 2024).

A central element of the KPH breeding program is the evaluation of young animals in FPTs, which places the optimal age for introducing a horse to training in the context of considerable discussion and controversy. The scientific literature presents numerous arguments both for and against early conditioning (Lewczuk, 2015). A key factor in this debate is musculoskeletal development, because bone adaptation to training is a highly individual process influenced by various factors (Firth et al., 1999; Firth & Rogers, 2005; Firth, 2006; Verheyen et al., 2006). While severe limb injuries, such as fractures, are often attributed to acute accidents (Stover, 2003; Wilk et al., 2020), other conditions, such as periostitis, can result from overloading. Adaptation of the skeleton, joints, and ligaments to training loads is not uniform, which underscores the need for a gradual and systematic training program (Firth, 2006).

Current industry practices reflect this variability. For instance, 83% of Warmblood horses begin their competition careers between four and six years of age (Rovere et al., 2017; Viklund, Blom & Eriksson, 2026). In contrast, Thoroughbreds and Purebred Arabians may start racing as three-year-olds. These examples suggest that work under saddle commonly begins at about three years of age, and even earlier in Thoroughbreds (Logan & Nielsen, 2021), when a horse’s potential for disciplines such as dressage may initially be assessed on the basis of movement (Barrey et al., 2002; Novotna et al., 2022). Performance tests are also tailored to specific breed types, as exemplified by the Hucul horse, another primitive breed with a pony-type conformation. Young Huculs undergo a specialized test known as the ‘Hucul path,’ a course of field obstacles designed to evaluate the horse’s responsiveness and willingness to cooperate with a rider (Stachurska et al., 2006; Krupa & Topczewska, 2012; Topczewska et al., 2021).

The appropriate training load relative to age has become a growing concern in recent years. Two primary factors drive this concern. First, as the average life expectancy of horses increases, there is greater emphasis on ensuring a long, healthy, functional lifespan (Lewczuk, 2015). Second, there is rising societal pressure from animal welfare organizations, which advocate for the horse’s role as a companion animal and question traditional uses such as riding, harness work, and slaughter. In this context, the goal of functional selection within the KPH breed is to produce an individual with a conformation suitable for recreational use. Along with the desire to use well-trained, willing KPHs under saddle, there is also a growing need to improve the efficiency of the horses’ movement, especially stride length at the walk and trot. Key selection criteria include functional build, correct movement, efficient gaits, willingness to work, and a gentle disposition toward humans.

Therefore, the aims of this study were to analyze: (1) how walk and trot performance in KPHs has changed over the past 25 years of field testing; (2) whether significant performance differences exist among birth cohorts; (3) the effect of age at testing on the recorded results; and (4) whether walk and trot performance are hereditary traits or, in this horse population, are shaped primarily by appropriate training.

## Materials & Methods

### Study design

This study was designed as a retrospective analysis of performance data from the official Field Performance Tests (FPTs) for Konik Polski horses, spanning the years 2000 to 2024. The data, consisting of historical records from these routine evaluations, were systematically recorded and cataloged by the official judges conducting each test. The methodological approach was therefore observational, using official records from the Konik Polski Horse Breeders Association to assess long-term performance trends without experimental intervention.

The experimental unit was the individual horse in a single test event. The study design focused on evaluating the effects of three fixed factors on performance: test type (riding or harness), calendar year of the test, and horse age at testing. The primary response variables were quantitative metrics of gait efficiency measured consistently throughout the 25-year period: specifically, stride length (cm) at the walk and trot and the time (s) required to cover 500 m at the walk and trot. These variables were selected to ensure consistency of the longitudinal analysis and to avoid confounding effects from historical modifications to the FPT protocol. A detailed description of the test protocol and its evolution over the study period is provided below.

### Field performance test protocol

#### Evaluated elements and scoring

Each horse was evaluated in one of two mutually exclusive test types: a riding test conducted under saddle or a harness test. The basic FPT protocol for both test types consisted of eight evaluated elements designed to provide a comprehensive assessment of horse utility (Table 1):

**Table 1:** Comparision of field performance tests for konik polski horses.

Parameter	Test variant	Protocol (2000–2019)	Protocol (from 2020)	Quantified change (Δ)
Total Distance	Saddle	9,000 m	3,600 m	−60%
	Harness	8,000 m	3,200 m	−60%
Protocol Structure	Saddle	5 sections × (600 m walk + 600 m trot + 600 m canter)	2 repetitions × (600 m walk + 600 m trot + 600 m canter)	Reduction from 5 to 2 cycles
	Harness	5 sections × 800 m (alternating walk/trot)	2 sections × 800 m (alternating walk/trot)	Reduction from 5 to 2 cycles
Temporal Duration (per unit)	Saddle	Walk: 8 min, Trot: 3 min, Canter: 1 min (Total: 12 min/section)	Walk: 7 min, Trot: 3 min, Canter: 1 min (Total: 11 min/rep)	Walk phase reduced by 1 min; Total time reduced by ∼63%
	Harness	Walk: 9 min, Trot: 3 min (Total: 12 min/section)	Walk: 8 min, Trot: 3 min (Total: 11 min/rep)	Walk phase reduced by 1 min; Total time reduced by ∼63%
Physiological Assessment	Both	Measurement at T+30 min post-exercise	Measurement at T+15 min post-exercise	50% reduction in recovery time window
Evaluated Traits (*n* = 8)	Both	1. Behavior, 2. Walk Speed, 3. Trot Speed, 4. Walk Stride Length, 5. Trot Stride Length, 6. Endurance, 7. Heart Rate, 8. Respiration Rate	No change in trait selection	None (Trait stability maintained)
Scoring Criteria	Both	0–5 points per trait (Max: 40 points)	0–5 points per trait (Max: 40 points)	None
Qualification Threshold	Both	Minimum: 21 points (52.5%)	Minimum: 21 points (52.5%)	None

1. Behavior: A subjective assessment of the horse’s temperament and trainability, including its conduct during saddling or harnessing, obedience to aids, and overall cooperation throughout the trial.
2. Walk Speed: Measured as the time taken to cover a 500 m distance, providing a quantitative measure of velocity at the walk.
3. Trot Speed: Measured as the time taken to cover a 500 m distance, providing a quantitative measure of velocity at the trot.
4. Walk Stride Length: Calculated based on the number of strides counted over a 100 m segment to quantify the efficiency and ground-cover of the walk.
5. Trot Stride Length: Calculated based on the number of strides counted over a 100 m segment to quantify the efficiency and ground-cover of the trot.
6. Exercise Test: Designed to assess stamina and endurance, this test was conducted in one of two formats:
  • Distance Variant: The time taken to complete a fixed distance was measured. For the harness test, the distance was 3,200 m (two repetitions of an 800 m walk and 800 m trot cycle). For the riding test, the distance was 3,600 m (two repetitions of a 600 m walk, 600 m trot, and 600 m canter cycle).
  • Time Variant: The total distance covered within a set time was measured. For the riding test, there were two repetitions of a cycle consisting of a 7-minute walk, a 3-minute trot, and a 1-minute canter. The harness test consisted of two repetitions: an 8-minute walk and a 3-minute trot cycle.
7. Heart Rate Recovery: The difference between the resting heart rate measured before the test and the rate recorded 15 min post-exercise, serving as an indicator of cardiovascular fitness.
8. Respiration Rate Recovery: The difference between the resting respiration rate and the rate recorded 15 min post-exercise, providing an additional measure of physiological recovery.

For each of the eight elements, horses could receive a maximum of 5 points, leading to a maximum total score of 40 points. A minimum of 21 points was required to pass the test officially. Based on the total score, a horse’s performance was classified as: sufficient (21–25 points), good (26–30), excellent (31–35), or outstanding (36–40). This scoring system provided a standardized, quantitative assessment of each horse’s overall performance.

#### Historical changes to the protocol

While the eight core elements of the FPT remained consistent, the exercise test protocol was more demanding from 2000 to 2019. In the distance variant, the total distance was 8,000 m for the harness test and 9,000 m for the riding test. In the time variant, the exercise protocol consisted of five repetitions of the work cycles. Furthermore, physiological indicators (heart rate and respiration) were measured 30 min after test completion rather than 15 min afterward.

Over the past 25 years, the scoring criteria for the measurable elements have also become progressively stricter. This adjustment was implemented in response to observed improvements in the breed’s movement indices. It was intended to provide more precise differentiation among high-performing individuals, because the previous system often resulted in a high concentration of ‘excellent’ or ‘outstanding’ scores that did not fully reflect the spectrum of abilities.

#### Limitations of the study

Several limitations inherent to the study’s retrospective design should be acknowledged. Factors such as rider or driver skill and weight, variation in carriage weight in the harness test, and differing environmental conditions across multiple field locations (*e.g.*, ground surface and weather) were not recorded in the dataset and could have contributed to the observed variability in performance. Finally, the FPT provides only a single performance snapshot early in a horse’s life. The data do not capture the effects of longer-term training, career longevity, or future suitability for recreational use, all of which are essential components of a horse’s overall value. Currently, the standard method for predicting genetic value is the BLUP statistical method combined with a biological model generally referred to as an animal or individual model. This method is commonly used to evaluate Warmblood horses that undergo, for example, 60- or 100-day training at a specific stud farm or stallion station in a given country. Thus, the procedure for predicting genetic value requires identification of the environment in which performance was achieved (*e.g.*, year and training facility). In the case of performance tests for KPHs, it was not possible to identify the training facilities, and the tests were conducted at various locations depending on the year of testing (ranging from several to 19 locations per year).

### Statistical analysis

All computations were carried out using Python (version 3.11) with the pandas (2.2), SciPy (1.11), scikit-posthocs (0.7), seaborn (0.13), and Matplotlib (3.8) libraries. Continuous traits were initially examined for normality using graphical methods and the Shapiro–Wilk test. Because several distributions deviated from normality and variances were heterogeneous across classes, subsequent analyses were conducted using nonparametric rank-based procedures.

To compare performance across groups defined by the fixed factors (year, age, and test type), a Kruskal–Wallis one-way ANOVA was used. When an overall test was significant (*p* < 0.05), pairwise contrasts were explored with Dunn’s test, using a Bonferroni adjustment for multiple comparisons. To identify the specific year at which a generational shift in performance became statistically nonsignificant, a sliding-threshold scan was applied, evaluating successive birth cohorts with Dunn’s test and Holm-corrected *p*-values.

To investigate relationships over time and among the measured performance metrics, correlation analyses were performed. Temporal trends were quantified using Spearman’s rank correlation (*ρ*) between each trait and the calendar year or year of birth. The same statistic was used to assess monotonic age effects. The strength of association among all performance traits was examined using Spearman correlation matrices. For all tests, two-tailed *p*-values are reported to three decimal places, with values < 0.001 denoted as *p* < 0.001. Results are visualized using point plots showing group means ± 95% confidence intervals and heatmaps of correlation matrices displaying *ρ* values in the lower triangle and exact *p*-values in the upper triangle.

In addition to the nonparametric trend analysis, genetic parameters for the four quantitative performance traits (walk stride length, trot stride length, walk time, and trot time) were estimated using linear mixed models (LMMs). To estimate heritability (*h*^2^), a single-trait sire model was fitted. The sire model included the fixed effects of test type (riding or harness), sex, age at testing (years), and year of testing. Sire was included as a random effect. Sires with fewer than three progeny in the dataset were excluded from the heritability estimation. Heritability (*h*^2^) was subsequently calculated from the variance components as:

\begin{eqnarray*}{h}^{2}= \frac{ \left( 4{\sigma }_{s}^{2} \right) }{ \left( {\sigma }_{s}^{2}+{\sigma }_{e}^{2} \right) } \end{eqnarray*}

where ${\sigma }_{s}^{2}$ is the variance component of the sire and ${\sigma }_{e}^{2}$ is the residual (error) variance component.

Second, to quantify the proportion of variance attributable to the genealogical line, a separate single-trait LMM was fitted. The LMM for genealogical line included the same fixed effects as the sire model, but genealogical line was included as the sole random effect. The intraclass correlation coefficient (ICC) was calculated to represent the proportion of total variance explained by the genealogical line:

\begin{eqnarray*}ICC= \frac{ \left( {\sigma }_{l}^{2} \right) }{ \left( {\sigma }_{l}^{2}+{\sigma }_{e}^{2} \right) } \end{eqnarray*}

where ${\sigma }_{l}^{2}$ is the variance component of the genealogical line. Genealogical lines represented by fewer than five observations were excluded from the ICC estimation.

## Results

The retrospective analysis was based on 1,608 records, of which 1,161 related to horses taking the riding test and 447 to those taking the harness test (Table 2). A total of 1,090 horses passed the riding test, and 429 passed the harness test. 71 horses presented under saddle were disqualified. In the case of the driving test, 18 horses were disqualified.

**Table 2:** The number of horses taking part in field performance tests, divided into mares and stallions.

Year	Localisation (n)	Number of horses attended (n)	Passed (n)	Disqualification (n)/(%)	Mares (n)/(%)	Stallions (n)/(%)	Harness test (n)/(%)	Riding test (n)/(%)
2000	2	9	9	0	5	4	5	4
2001	2	10	9	1/(11,11)	6	4	4	6
2002	3	20	20	0	13	7	9	11
2003	3	13	13	0	5	8	2	11
2004	4	24	22	2/(9,09)	16	8	3	21
2005	4	16	15	1/(6,66)	15	1	12	4
2006	5	26	26	0	12	14	6	20
2007	7	32	32	0	15	17	6	26
2008	4	23	23	0	11	12	6	17
2009	7	39	38	1/(2,63)	13	26	13	26
2010	9	44	44	0	19	25	15	29
2011	6	36	34	2/(5,88)	13	23	14	22
2012	4	28	28	0	14	14	4	24
2013	3	21	21	0	12	9	6	15
2014	2	14	14	0	8	6	5	9
2015	2	11	11	0	7	4	1	10
2016	3	18	18	0	13	5	1	17
2017	3	34	33	1/(3,03)	27	7	8	26
2018	8	73	73	0	62	11	12	61
2019	14	127	121	6/(4,95)	112	15	40	87
2020	11	113	108	5/(4,62)	98	15	31	82
2021	17	176	170	6/(3,52)	163	13	65	111
2022	17	178	164	14/(8,53)	169	9	64	114
2023	23	273	246	27/(10,97)	253	20	74	199
2024	20	250	227	23/(10,13)	225	25	41	209
Total	183	1,608	1,519	89/(5,53)	1,306/(81,21)	302/(18,78)	447/(27,80)	1,161 (72,20)

### Stride length in the walk during riding and harness tests

#### Riding test

**Figure 1:**
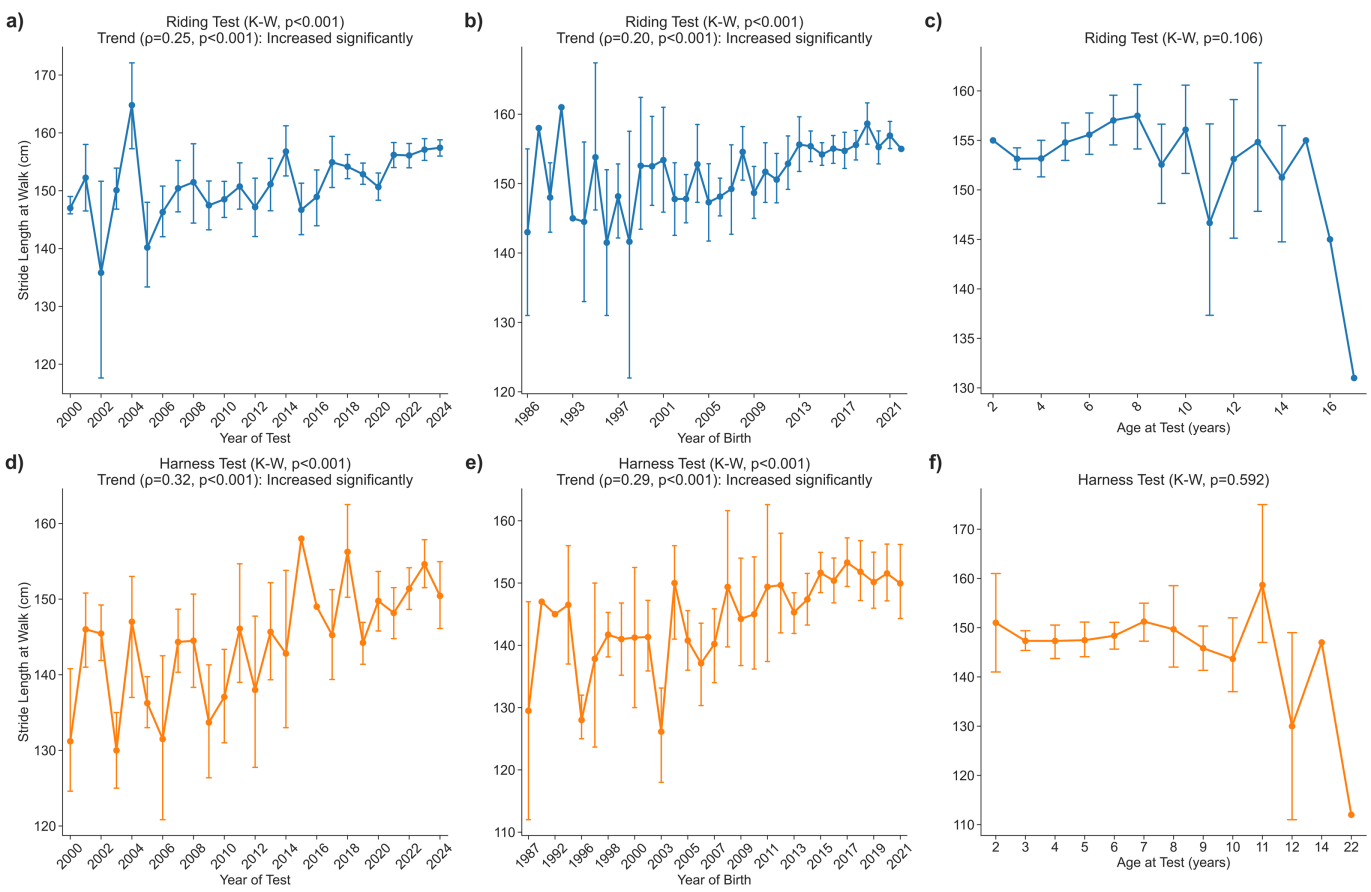
Stride length walk.

Stride length under saddle increased significantly with both the calendar year of testing (*p* < 0.001) and the horses’ year of birth (*p* < 0.001). Mean walk stride length increased from 147 ± 2 cm in 2000 to 157 ± 10 cm in 2024 (Fig. 1A).

A sliding-threshold *post-hoc* scan of consecutive birth years showed that 2011 was the last birth year for which the ≤ 2011 cohort retained a significantly shorter stride (149.9 ± 13.4 cm; *n* = 286) than horses born later (155.6 ± 11.1 cm; *n* = 818, Holm-adjusted *p* < 0.05). The small samples from the late-1980s and mid-1990s cohorts (≤ 11 horses each) did not differ significantly after this correction was applied, indicating that the generational improvement is best described as a broad shift beginning with foals born in 2012 (Fig. 1B).

Across the full age range represented (2–18 years), stride length was not influenced by the age at testing (*p* = 0.106), confirming the absence of an age effect (Fig. 1C).

#### Harness test

Stride length in harness also increased significantly with both the calendar year of testing (*p* < 0.001) and the horses’ year of birth (*p* < 0.001). The mean value rose from 131 ± 10 cm in 2000 to 150 ± 14 cm in 2024 (Fig. 1D).

A sliding-threshold *post-hoc* scan of consecutive birth years identified 2017 as the last birth year for which the ≤ 2017 cohort retained a significantly shorter stride (146.8 ± 13.0 cm; *n* = 304) than horses born later (151.1 ± 13.9 cm; *n* = 116, Holm-adjusted *p* < 0.05) (Fig. 1E).

Across the entire age span represented (3–18 years), stride length was unaffected by age at testing (*p* = 0.565) (Fig. 1F).

### Stride length in the trot during riding and harness tests

#### Riding test

**Figure 2:**
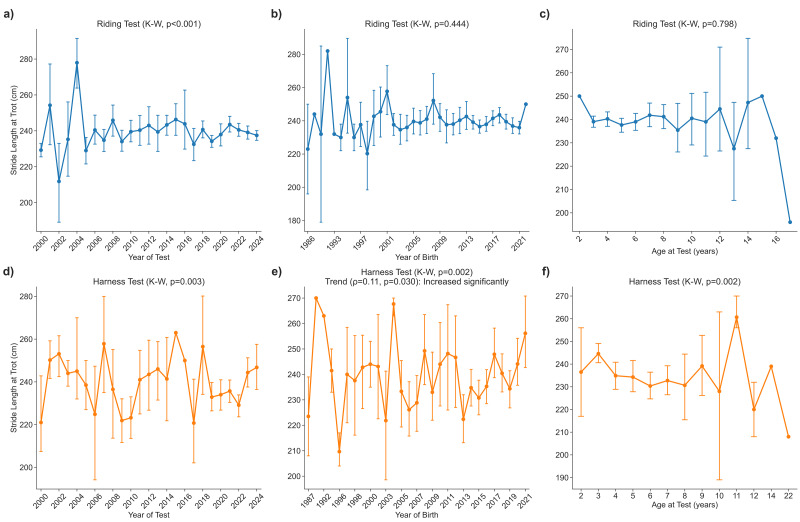
Stride length trot.

Stride length at the trot differed significantly among calendar years of testing (*p* < 0.001); however, no consistent directional trend over time was observed. The mean increased from 228 ± 14 cm in 2000 to 238 ± 19 cm in 2024 (Fig. 2A). A sliding *post-hoc* scan revealed no birth-year threshold after Holm correction (all *p* ≥ 0.05) (Fig. 2B). Across the full age range (2–18 years), trot stride length remained age-independent (*p* = 0.798) (Fig. 2C).

#### Harness test

Stride length at the trot also differed significantly among calendar years of testing (*p* = 0.003), but again, no clear temporal trend was detected. The mean rose from 221 ±24 cm in 2000 to 247 ± 32 cm in 2024 (Fig. 2D).

Stride length in harness varied significantly with year of birth (*p* = 0.002) and showed a positive association with birth year (*p* = 0.030). A sliding threshold analysis identified 2017 as the last significant split: horses born ≤ 2017 averaged 239 ± 22 cm (*n* = 308), whereas those born after 2017 averaged 253 ± 19 cm (*n* = 116; Holm-adjusted *p* < 0.05), indicating a generational improvement beginning with the 2018 cohort (Fig. 2E). At three years of age, the mean trot stride length was 245 ± 28 cm, with a decreasing trend observed in older age classes: 235 ± 24 cm (4 years), 234 ± 24 cm (5 years), 230 ± 25 cm (6 years), 233 ± 23 cm (7 years), and 231 ± 31 cm (8 years) (Fig. 2F).

### Time to cover 500 m at a walk during riding and harness tests

#### Riding test

**Figure 3:**
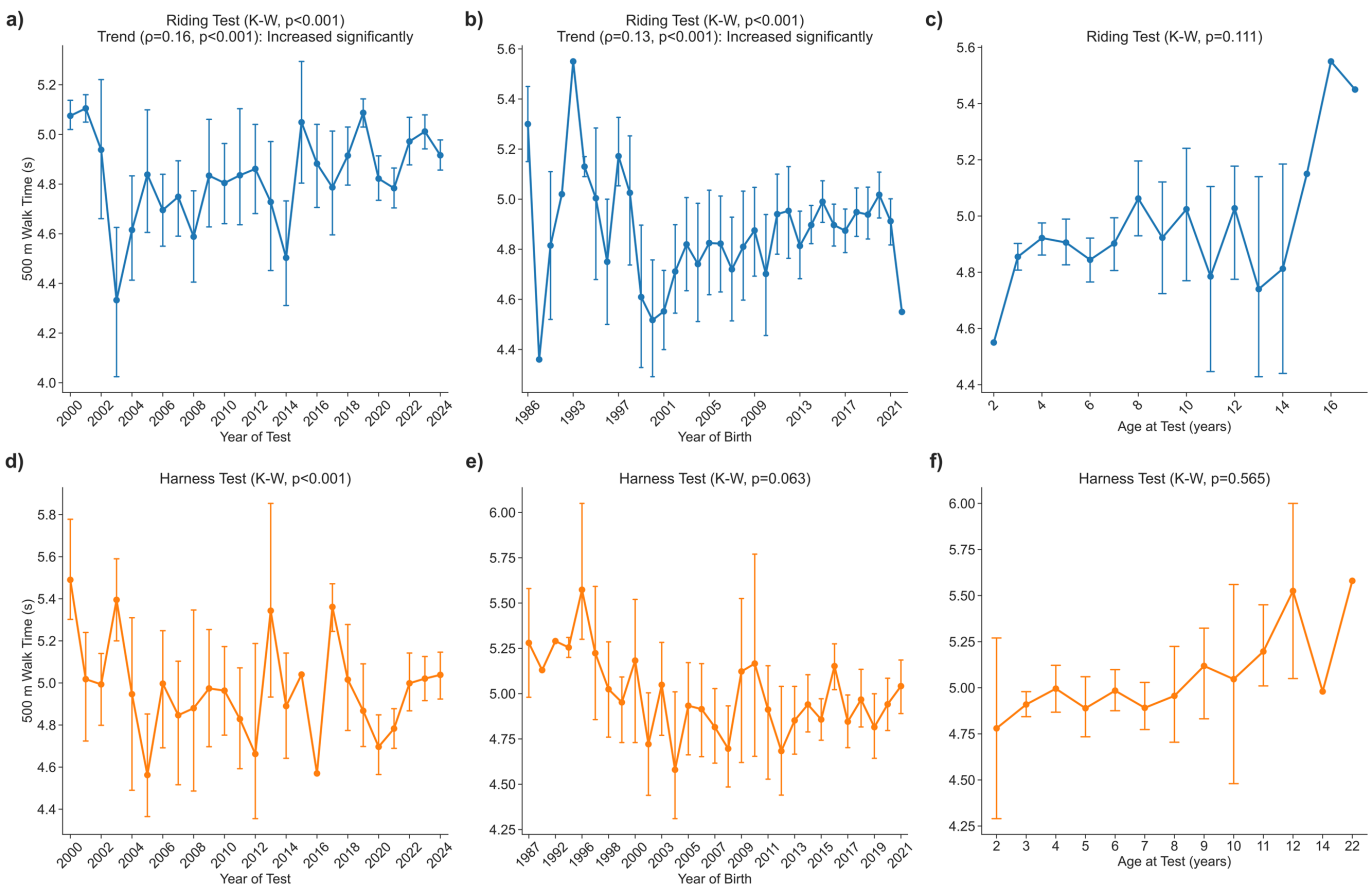
Time walk 500 m.

In the riding test, the time required to walk 500 m increased slightly over the past 25 years. A moderate positive association with the calendar year of testing was found (*p* < 0.001), which was mirrored by a weaker but still significant relationship with the year of birth (*p* < 0.001). The fastest cohort was recorded in 2003, averaging 260 ± 33 s, whereas the slowest was recorded in 2001 at 306 ± 4 s; by 2024, the mean had decreased to 295 ± 26 s. Walking time remained age-independent across the full 2–18 year range (*p* = 0.111) (Figs. 3A–3C).

#### Harness test

In the harness test, no consistent temporal or generational trend emerged; only significant year-to-year differences were detected (*p* < 0.001). Walking time showed no significant correlation with either the year of testing (*ρ* = 0.06, *p* = 0.256) or the year of birth (*ρ* ≈ 0.00, *p* = 0.960). The fastest mean occurred in 2005 at 274 ± 18 s, the slowest in 2000 at 329 ± 19 s, and the 2024 cohort averaged 302 ± 21 s. Age had no discernible effect (*p* = 0.565), indicating that walking speed in harness remained stable across years, birth cohorts, and ages (Figs. 3D–3F).

### Time to cover 500 m at a trot during riding and harness tests

#### Riding test

**Figure 4:**
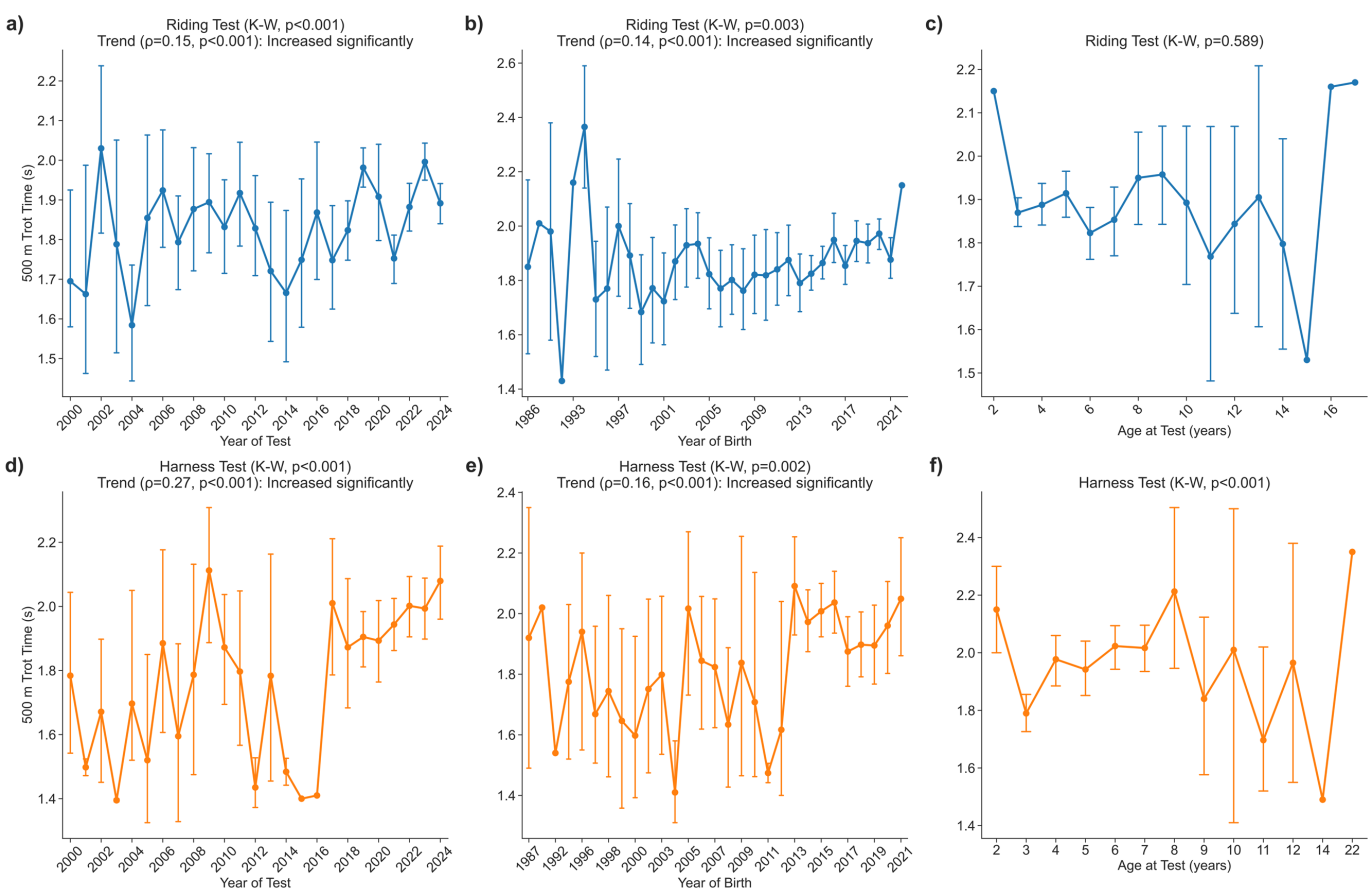
Time trot 500 m.

The time required to trot 500 m increased significantly over the 25 years analyzed (*p* < 0.001), with mean values rising from 102 ± 14 s in 2000 to 113 ± 21 s in 2024 (Fig. 4A). A positive relationship with year of birth was also apparent (*p* < 0.001); the 1999 cohort was fastest at 101 ± 24 s, whereas the 1994 cohort was slowest at 142 ± 19 s (Fig. 4B). Trotting time did not vary by age (*p* = 0.589) across the full 2–18-year range (Fig. 4C).

#### Harness test

Trotting time in harness also lengthened significantly (*p* < 0.001), climbing from 107 ± 19 s in 2000 (*n* = 5) to 125 ± 22 s in 2024 (*n* = 37) (Fig. 4D). A marked generational effect was present (*p* < 0.001): horses born in 2004 were quickest at 85 ± 9 s (*n* = 3), whereas the 2013 cohort was slowest at 125 ± 24 s (*n* = 23) (Fig. 4E). Age influenced performance (*p* < 0.001); three-year-olds averaged 107 ± 24 s, and times increased progressively with age, reaching 122 ± 19 s in seven-year-olds (Fig. 4F).

### Correlations among performance parameters during riding and harness tests

#### Riding test

**Figure 5:**
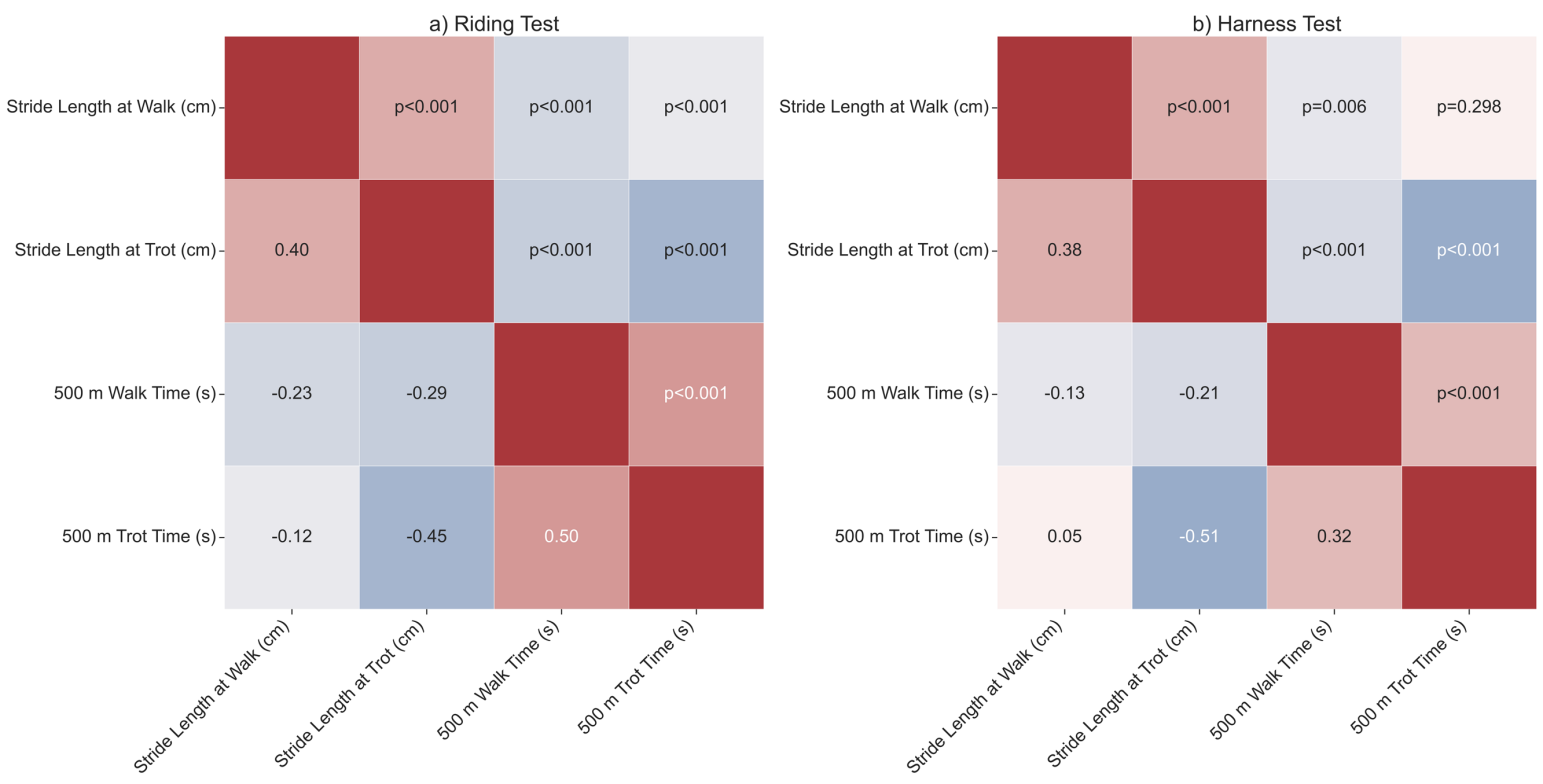
Correlation matrix.

In the riding tests, the two speed measures were strongly correlated; horses that took longer to cover 500 m at the walk also took longer at the trot (*ρ* = 0.50, *p* < 0.001). Stride length traits followed a similar pattern, with longer strides at the walk accompanying longer strides at the trot (*ρ* = 0.40, *p* < 0.001). Faster trotting times were achieved mainly through longer strides, as shown by a moderate negative correlation between trot stride length and trot time (*ρ* = −0.45, *p* < 0.001). Weaker inverse relationships were also observed between walk stride length and both walk time (*ρ* = −0.23, *p* < 0.001) and trot time (*ρ* = −0.12, *p* < 0.001). Similarly, trot stride length was inversely related to walk time (*ρ* = −0.29, *p* < 0.001) (Fig. 5A).

#### Harness test

Patterns in the harness tests were comparable but slightly less pronounced. The two speed measures were positively related (*ρ* = 0.32, *p* < 0.001), and stride lengths at the walk and trot again covaried (*ρ* = 0.38, *p* <  0.001). As under saddle, longer strides at the trot were associated with faster completion times, evidenced by a strong negative correlation (*ρ* = −0.51, *p* < 0.001). Trot stride length also showed a weaker inverse link with walk time (*ρ* = −0.21, *p* < 0.001). Walk stride length was only modestly related to walk time (*ρ* = −0.13, *p* = 0.006) and showed no significant association with trot time (*ρ* = 0.05, *p* = 0.298) (Fig. 5B).

### Genetic parameter estimates

The sire model for heritability estimation used 1,377 valid performance records from 184 unique sires. The analysis revealed moderate heritability for walk stride length (*h*^2^ = 0.277). The sire variance component (${\sigma }_{s}^{2}$) for this trait was estimated at 9.543, whereas the residual variance (*σ*_*e*_^2^) was 128.248.

In contrast, the heritability estimates for the other three traits—trot stride length, walk time over 500 m, and trot time over 500 m—were negligible (*h*^2^ = 0). The model did not detect a significant sire variance component for these traits, attributing the variation primarily to residual effects (Table 3).

**Table 3:** Heritability estimates (*h*^2^) and variance components from the sire model.

Trait	Sire variance (${\sigma }_{s}^{2}$)	Residual variance (${\sigma }_{e}^{2}$)	Heritability (*h*^2^)
Walk Stride Length (cm)	9.543	128.248	0.277
Trot Stride Length (cm)	0	417.766	0
Walk Time (s)	0	0.156	0
Trot Time (s)	0	0.092	0

The analysis of genealogical line variance, comprising 1,516 observations from 22 distinct lines, revealed that genealogical lines contributed a negligible proportion of the total phenotypic variance for most traits. The ICC was estimated at 0.000 for walk stride length, trot stride length, and walk time. A minor nonzero variance component was detected for trot time, with the genealogical line explaining 1.4% of the observed variation (ICC = 0.014) (Table 4).

**Table 4:** Intraclass Correlation Coefficient (ICC) and variance components for pedigree line.

Trait	Line variance (${\sigma }_{s}^{2}$)	Residual variance (${\sigma }_{e}^{2}$)	ICC (variance %)
Walk stride length (cm)	0	132.951	0.000 (0.0%)
Trot stride length (cm)	0	513.744	0.000 (0.0%)
Walk time (s)	0	0.198	0.000 (0.0%)
Trot time (s)	0.002	0.125	0.014 (1.4%)

## Discussion

Our 25-year retrospective analysis of FPTs for Konik Polski horses provides the first long-term evidence of performance trends in this primitive breed. The primary finding was a significant and consistent improvement in walk stride length in horses subjected to both riding and harness tests, suggesting a positive response to the selection pressure applied since mandatory testing was introduced. However, when heritability estimates are taken into account, improvement in walk performance appears to depend mainly on appropriate training, as already noted by Tomczyński, Kopel & Jaworowska (1986) and Tomczynski et al. (1988). As the skills and awareness of those preparing horses for performance tests increase, the level of preparation and movement efficiency likely also improves. Improved gait efficiency was evident in the walk because this is the basic gait in primitive horses. The observed increase in walk stride length by nearly 10 cm under saddle is of considerable practical importance because it directly translates into greater gait efficiency and rider comfort, thereby increasing the breed’s value for its primary recreational use.

However, the performance testing system for KPHs differs substantially from the more intensive models commonly used for European Warmblood breeds. While major breeding associations for sport horses often rely on long-duration station tests, particularly for stallions (lasting up to 70 days) (Thorén Hellsten et al., 2006), KPH evaluation is based on a 1-day field test. This FPT model, used by some Warmblood organizations for mares and geldings (Novotna et al., 2022; Lewczuk et al., 2026), serves as the foundational method for both sexes in the KPH breeding program. The field-based approach is more accessible for breeders with a dispersed horse population and is less costly, but it may introduce greater environmental variability than highly standardized station tests. Despite this, the clear directional trend in stride length suggests that adequate selection pressure is present.

### Implications of protocol changes and breed-specific test design

A critical factor in interpreting the long-term data is the significant modification of the FPT protocol after 2019. The substantial reduction in the exercise test distance (*e.g.*, 9,000 m to 3,600 m in the riding test) fundamentally reduced the test’s demand on the horse’s endurance. This change in methodology must be taken into account when evaluating trends in other parameters. For instance, the concurrent finding that the time taken to cover 500 m at a trot has slightly increased over the 25 years may be partially explained by these protocol changes. With a less demanding final exercise, horses may not be conditioned to the same level of fitness as in earlier years, potentially affecting their speed in shorter, timed segments.

The higher endurance demands of the FPT protocol, which remained in effect until 2019, necessitated thorough physical preparation by breeders. The recent shortening of the test may have reduced this emphasis on conditioning, as reflected in the disqualification rate (7.55%) observed in the current dataset, primarily because of inadequate preparation. The design of the KPH test itself is fundamentally different from that used for Warmblood sport horses. While assessments for Warmbloods focus on elements crucial to elite sport, such as gait quality, dressage potential, and jumping ability, the KPH test omits these components. This distinction aligns with the breed’s primary purpose as a recreational riding horse, often for children, teenagers, and lighter adults, rather than as a high-performance sport horse. Consequently, KPH evaluation appropriately focuses on functional gait indicators, including walk and trot stride length and speed.

### Genetic trend and historical context of walk stride length

A key finding of our study is the systematic increase in walk stride length over the 25-year period, a favorable outcome consistent with the goals of the KPH breeding program. The average stride length under saddle in recent years reached 157 cm, which compares favorably with historical data. For instance, studies from the 1980s reported a range of 131–177 cm for KPHs under various loads, with an average of approximately 140–160 cm for unloaded horses (Jodkowska et al., 1984). Similarly, data from 2000 to 2006 showed a range of approximately 131–165 cm (Balińska, 2009). The current results therefore provide quantitative evidence of improvement in this trait. An assessment of heritability revealed that walk stride length is only moderately heritable (*h*^2^ = 0.277), whereas environmental factors shape it much more strongly. It can therefore be assumed that the improving stride-length values reflect deliberate training during preparation for the performance test. The other measured traits—trot stride length and speed over 500-m sections at the walk and trot—also appear to be shaped primarily by proper training rather than heredity. It is well known that performance traits, such as dressage results, typically exhibit low heritability because of strong environmental influence (Solé et al., 2014). Research on the heritability of locomotor traits has been conducted in Lusitano horses, whose population is approximately twice that of KPHs. Lusitanos are used in dressage, carriage driving, and working equitation. In the study by Solé et al. (2014), videographic measurements of kinematic features were used, although the analyses were limited to trotting. In contrast to the present results, trot stride length in Lusitano horses is a highly heritable trait (*h*^2^ = 0.49) (Solé et al., 2014). However, conscious breeding and selection aimed at improving movement efficiency in Lusitano horses have been underway for much longer than in KPHs. It must be acknowledged that in KPHs, selection for performance was historically limited, and stallions with more efficient movement were not preferentially promoted in breeding. In practice, all stallions that passed the test were used for breeding, even if their movement efficiency was poor. Only now, as KPHs are becoming increasingly popular as working horses, is this situation likely to change because riders will prefer horses with better movement efficiency. Therefore, it is worth emphasizing that deliberate breeding work to improve performance traits began in this breed only a few years ago. It is conceivable that, in two or three decades, if selection based on walking efficiency is consistently applied, this trait may become more clearly heritable.

Interestingly, this study found no significant effect of horse age at testing on walk stride length. This suggests that whether a horse is tested at three, four, or an older age, the level of preparation is likely the determining factor in performance rather than age-related physical maturity alone. It is plausible that most horses, regardless of age, are presented for the FPT after a similar, relatively short period of focused training, which would standardize performance across age groups.

### Variability in trot performance and the influence of training

In contrast to the apparent trend observed for the walk, trot stride length did not show consistent directional improvement over the 25-year period. However, a generational improvement was noted in the harness test for cohorts born after 2017. The data nevertheless revealed substantial variability in trot performance. The high degree of observed variation is consistent with previous research on the breed, which reported wide ranges in trot stride length, from 220 cm to 291 cm (Jodkowska et al., 1984; Sasimowski et al., 1989; Górecka et al., 2003; Balińska, 2009). Several unmeasured factors inherent to the field-based testing system may contribute to this variability. As FPTs are conducted in numerous locations, variation in ground surface can influence gait mechanics. Furthermore, in the harness test, inconsistency in carriage weight could explain the greater dispersion of results observed compared with the riding test.

Other factors, such as rider influence, cannot be ruled out. Although KPHs are typically ridden by young, light individuals, variation in rider weight and skill may still affect a horse’s movement, particularly at the trot, where the load on the back is greater. A more important factor, however, appears to be the duration and intensity of training. In a study by Tomczyński, Kopel & Jaworowska (1986), KPHs subjected to a year-long training program showed a substantial increase in trot stride length, averaging 17 cm. This contrasts sharply with typical preparation for the FPT, where most horses, regardless of age, undergo only 2–3 months of work before testing (Tomczyński, Kopel & Jaworowska, 1986).

The limited training period explains the counterintuitive finding that older horses did not outperform their three-year-old counterparts. In fact, in the harness test, three-year-olds achieved the longest average trot stride. This finding suggests that, in the current system, age at testing is not a reliable proxy for training duration. It is plausible that horses tested at a later age are just beginning work, as are three-year-olds. It can be speculated that if horses were presented for the FPT after a minimum of one year of consistent training, as demonstrated by Tomczyński, Kopel & Jaworowska (1986), they would likely achieve better results in both stride length and speed.

### Analysis of speed trends and trait correlations

In addition to stride length, the study analyzed the time taken to cover 500 m as an indicator of gait efficiency, a metric influenced not only by a horse’s physical capabilities but also by its willingness to move forward and responsiveness to aids. A concerning finding was that the time taken to complete 500 m at a trot increased over the 25 years in both riding tests (from 102 ± 14 s in 2000 to 113 ± 21 s in 2024) and harness tests (from 107 ± 19 s to 125 ± 22 s). The observed decline in speed, concurrent with an improvement in stride length, may be an unintended consequence of modifications to the FPT protocol. As discussed previously, shortening the endurance component of the test may have reduced breeders’ incentive to condition horses to higher levels of fitness. Support for this interpretation comes from research by Tomczynski et al. (1988), who demonstrated that a 10-month training program significantly improved both speed and stride length in KPH stallions.

The low heritability of individual walk and trot traits observed in all 22 genealogical lines, both female and male, indicates a much greater contribution of environmental factors, especially proper training, as suggested in earlier studies by Tomczyński et al. (1986), Tomczyński et al. (1988). In contrast, the FPT provides only a single snapshot of performance after a relatively short preparation period. A further limitation of the present study is the lack of longitudinal data tracking individual horses’ development or their competition performance, as many animals, particularly mares, are used solely for breeding after passing the FPT (Tomczynski et al., 1988).

The analysis also confirmed significant correlations among gait-efficiency indicators. A moderately positive correlation was found between walk and trot stride length in the riding (*ρ* = 0.40) and harness (*ρ* = 0.38) tests, suggesting a common biomechanical or genetic basis for gait quality. These correlations are consistent with, although lower than, the value reported by Górecka et al. (2003) (*ρ* = 0.75) in an earlier analysis of KPHs. The correlation also appears lower than that reported in Warmblood stallions, where values can reach 0.92 (Thorén Hellsten et al., 2006), likely reflecting the more intensive and targeted selection for gait quality in sport horse breeds. Crucially, a strong negative correlation was observed between trot stride length and the time taken to cover 500 m in both the harness test (*ρ* = −0.51) and the riding test (*ρ* = −0.45). From a breeding perspective, this is a highly desirable relationship because it indicates that faster horses achieved their speed through more efficient, longer strides rather than through rapid, shorter steps. This observation is consistent with previous research on KPHs, which also reported a significant negative correlation for the same relationship (*ρ* = −0.74) (Balińska, 2009).

### Strengths and limitations of the KPH testing model

The design and objectives of the KPH performance test differ from those for other primitive breeds, such as the Hucul horse. The “Hucul path” is considered a more comprehensive evaluation of a horse’s overall utility, encompassing its temperament and cooperation with the rider (Stachurska et al., 2006). In contrast, the strength of the KPH FPT lies in its focus on objective, highly repeatable metrics, such as time and stride length. While perhaps less holistic, this quantitative approach is particularly well-suited to retrospective analysis, as it provides measurable, objective data ideal for tracking changes in specific traits over decades. The importance of such testing is underscored by findings in Hucul horses, where the sire’s influence was a significant determinant of offspring performance, confirming that standardized evaluations are a critical tool for effective breeding work (Budzynska et al., 2003).

## Conclusions

Integrating performance data is essential for estimating reliable breeding values in KPHs. The reduction in the test’s endurance component is associated with a concerning decline in speed and may result in inadequate physical preparation of the tested horses. Movement parameters in KPHs appear to depend far more on environmental conditions than on hereditary factors, which underscores the importance of proper training in this breed. It is possible that in the future, through careful selection for functional traits, gait efficiency will become more clearly heritable. To make better use of FPT results in selection, future considerations could include modifications to the testing system or the reintroduction of stationary training facilities, particularly for stallions, to ensure more comprehensive evaluation and to continue the balanced improvement of this unique primitive breed. Despite these limitations, the study provides the first comprehensive long-term analysis of performance trends in the KPH breed, offering valuable insights for its conservation and breeding program.

## Supplemental Information

10.7717/peerj.21369/supp-1 Supplemental Information 1 Data on horses participating in performance tests and scores for individual test elements

## Additional Information and Declarations

### Data Availability

The following information was supplied regarding data availability: The raw measurements are available in the Supplementary File.
